# Rested-Baseline Responsivity of the Ventral Striatum Is Associated With Caloric and Macronutrient Intake During One Night of Sleep Deprivation

**DOI:** 10.3389/fpsyt.2018.00749

**Published:** 2019-01-17

**Authors:** Brieann C. Satterfield, Adam C. Raikes, William D. S. Killgore

**Affiliations:** ^1^Social, Cognitive, and Affective Neuroscience Laboratory, Department of Psychiatry, College of Medicine, University of Arizona, Tucson, AZ, United States; ^2^Department of Psychiatry, McLean Hospital, Harvard Medical School, Belmont, MA, United States

**Keywords:** sleep deprivation, ventral striatum, nucleus accumbens, food consumption, reward

## Abstract

**Background:** Sleep loss contributes to obesity through a variety of mechanisms, including neuroendocrine functioning, increased hunger, and increased food intake. Additionally, sleep loss alters functional activation within brain regions associated with reward and behavioral control. However, it remains unknown whether individual differences in baseline neural functioning can predict eating behaviors during total sleep deprivation (TSD). We used functional magnetic resonance imaging (fMRI) to test the hypothesis that individuals with increased baseline responsiveness within reward regions are more vulnerable to TSD-induced overeating.

**Methods:** *N* = 45 subjects completed several fMRI scans during a single pre-TSD session that included performance on the Multi-Source Interference Task (MSIT) and the *n-*back task. Subjects returned to the laboratory for an overnight TSD session, during which they were given *ad libitum* access to 10,900 kcal of food. Leftover food and packaging were collected every 6 h (00:00, 06:00, and 12:00) to measure total food consumption. Subjects reported sleepiness every hour and performed a food rating task every 3 h.

**Results:** Functional activation within the ventral striatum during the MSIT and *n*-back positively correlated with total caloric and carbohydrate intake during the final 6 h (06:00–12:00) of TSD. Activation within the middle and superior temporal gyri during the MSIT also correlated with total carbohydrates consumed. Food consumption did not correlate with subjective sleepiness, hunger, or food desire.

**Conclusions:** Individual differences in neural activity of reward processing areas (i.e., nucleus accumbens) *prior to* sleep deprivation are associated with an individual's propensity to overeat during subsequent sleep deprivation. This suggests that individual differences within reward processing pathways are potential key factors in sleep loss related overeating. Sleep loss and obesity are tightly linked. Both phenomena have been associated with increased neural activation in regions associated with reward, inhibitory control, and disrupted dopamine signaling. Elevated baseline reward sensitivity in the ventral striatum appears to be further compounded by sleep deprivation induced dysfunction in the reward neurocircuitry, increasing the likelihood of overeating. Our findings suggest that large individual differences in baseline responsiveness of hedonic reward pathways may modulate the association between sleep loss and obesity.

## Introduction

The social and economic demands of living in our modern 24/7 society have contributed to two pervasive problems: insufficient sleep and obesity (1). The National Sleep Foundation recommends that adults obtain ≥7 h of sleep per night (2). However, 35% of adults in the United States sleep <7 h per night (3), a nightly duration that has been on the decline for the last several decades. Simultaneously, obesity rates have dramatically increased, with over one third of the adult population being classified as obese (4). Epidemiological evidence suggests a strong association between the declining levels of sleep and increasing rates of obesity (1, 5–7).

Sleep loss contributes to weight gain through several physiological, behavioral, and neural mechanisms. From a physiological perspective, sleep loss disrupts the normal functioning of hormones that regulate appetite (ghrelin) and satiety (leptin). Studies have demonstrated that sleep restriction results in elevated levels of ghrelin and reduced levels of leptin, leading to increased feelings of hunger (8–11). Behavioral studies of sleep restriction and sleep deprivation have also demonstrated how sleep loss impacts eating behaviors. Sleep loss leads to increased energy intake, while energy expenditure does not change (12, 13), contributing to overall weight gain (14). Individuals tend to increase their overall total caloric intake (13–16), especially in the form of carbohydrates (16) and fat (13) during sleep loss. These extra calories come from snacks (17, 18) and increased meal frequency (14).

While studies have focused on how sleep loss disrupts neuroendocrine signaling and modifies eating behavior, few have investigated the brain's neural response to food stimuli. There are several interacting neural networks which drive food intake behavior, including connections between several key cortical [orbital frontal cortex (OFC), prefrontal cortex (PFC), insula, and anterior cingulate cortex (ACC)] and limbic (amygdala, hippocampus, and basal ganglia) regions (19–22). Volkow et al. (22) suggest that obesity may be the result of an imbalance between neural circuits that promote eating behaviors (reward-saliency and motivation-drive circuits) and those that control and inhibit behavioral responses (learning-condition and inhibitory control-emotion regulation circuits) (22). Sleep loss also disrupts communication between cortical regulatory and subcortical reward systems. There is elevated neural activity in regions associated with reward and risky decision-making and attenuated activity in cortical regions associated with inhibitory control (23–25). The parallels in altered signaling patterns in these key systems for both obesity and sleep loss suggest that both conditions result in a loss of top-down inhibitory control over reward-processing regions.

Studies investigating the neural responses to food stimuli associated with daytime sleepiness, sleep restriction, and total sleep deprivation (TSD) have found evidence supporting a disruption of behavioral control and reward systems (21, 26–30). Excessive daytime sleepiness is associated with reduced neural activation in the ventromedial PFC (vmPFC), an area involved in inhibitory control, when viewing images of high calorie foods. Reduced activation in this region also correlates with subjective difficulty restricting food consumption (21). Lack of inhibition from the frontal control system may release a “brake” on subcortical pathways involved in modulating reward-based behaviors, such as eating.

Sleep restriction also impacts neural activity in regions associated with reward, including the nucleus accumbens (NAc), putamen, and vmPFC. Neural activation within these regions tends to increase when viewing food items, further supporting the notion that sleep loss alters normal reward processing and inhibitory control (27, 31). Similarly, viewing images of calorie-rich unhealthy food increases activity in regions associated with hedonic eating (i.e., eating for pleasure), including the frontal, temporal, and parietal cortices, as well as the OFC and insula (30). Activity in the inferior frontal gyrus, a region associated with inhibitory control, has also been found to increase in response to food cues (27). In addition to the regions described above, several avenues of research have found functional activation and connectivity changes in areas of the salience network [i.e., ACC, insula, etc. (32)]. This network is involved in identifying homeostatically relevant stimuli and evaluating and selecting valued options, such as food (26, 33, 34). The ACC also makes efferent and afferent connections with regions involved in reward pathways (33). Benedict et al. (26) were the first to investigate neural responses to food stimuli during TSD, finding that one night of sleep loss resulted in increased neural activation in the ACC (26). Further, one night of TSD enhanced functional connectivity between regions of the salience network, including the dorsal ACC and putamen, in relation to total fat consumption (28). Findings within the ACC suggest that sleep loss may modulate the desire for and rewarding properties of food, thus increasing the likelihood of overconsumption. Greer et al. (29) found that one night of sleep loss resulted in decreased neural activity in appetite evaluation regions (ACC, PFC, insula) and increased activation in the amygdala, further supporting the notion of reduced inhibitory control and increased reward drive during sleep loss (29).

Strong evidence points to altered functional activation within behavioral control and reward systems as one mechanism underlying the propensity to overeat during sleep loss. However, little work has focused on how individual differences in baseline neural activity within these circuits are associated with food consumption throughout a period of TSD. Individual differences in reward sensitivity are associated with the brain's response to food stimuli under rested conditions (19). Reward drive, as assessed by questionnaire, correlates with increased neural activation in regions of the fronto-striatal-midbrain reward circuitry (19). This hyper-responsivity of the reward network leads to an increased vulnerability to overeat. Here, we used functional magnetic resonance imaging (fMRI) to investigate how differences in pre-TSD functional activation within reward-related neural circuits can predict an individual's propensity to increase caloric and macronutrient intake during sleep loss. Specifically, in light of the well-established deficits in prefrontal inhibitory control during sleep deprivation (24, 35), we hypothesized that baseline hyper-activation in regions related to reward drive (e.g., ventral striatum) would be associated with an increased tendency to overeat during a subsequent period of sleep deprivation.

## Methods

### Subjects

Forty-five healthy adults (20–45 y; 22 females) participated in this three-part study conducted in the McLean Hospital Sleep Research Laboratory. Subjects eligible for study participation met the following criteria: free from sleep, psychological, neurological or other medical disorders; right-handed as assessed by the Edinburgh Handedness Inventory (36); primary English speakers; no vision impairment, unless corrected to normal with contact lenses; no drug or alcohol abuse in the past 6 months; no history of smoking or tobacco use in the past year; and no contraindications for neuroimaging, including pregnancy or metal in the body. In order to control for fluctuations in menstrual hormones which could directly impact performance and brain neurochemistry, females underwent the functional neuroimaging scan during the follicular phase of their menstrual cycle or were taking monophasic contraceptives. Female subjects were excluded if they used multiphasic birth control.

All study procedures were approved by the Institutional Review Board (IRB) of McLean Hospital and the United States Army Human Research Protection Office (HRPO). All subjects gave written informed consent in accordance with the Declaration of Helsinki and were financially compensated for their time.

### Experimental Design

**Figure 1:**
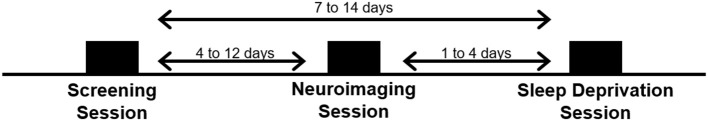
Schematic of the three experimental visits for the study.

Subjects visited the laboratory on three separate occasions: a screening session, a neuroimaging session, and a TSD session. See Figure 1.

#### Screening Session

Subjects first visited the lab for a comprehensive screening session. All study procedures were explained and subjects completed a series of questionnaires, including a brief psychiatric evaluation using the Mini International Neuropsychiatric Interview [MINI; (37)] to further confirm eligibility. Subjects were fitted with a wrist activity monitor (Actiwatch-2, Philips Respironics, Bend, OR) to track at-home sleep and wake patterns for at least 1 week (10.8±3.3 days) prior to the third visit (i.e., the TSD session). Subjects also completed a daily online sleep diary during this time.

#### Neuroimaging Session (Pre-TSD)

Subjects returned to the lab ~1 week (8.4 ± 3.2 days) following the initial screening session for a second visit. A 2 h afternoon neuroimaging session was conducted to collect structural and functional images while subjects performed a series of neurobehavioral tasks, including the Multi-Source Interference Task (MSIT) and the *n*-back task, which are described in detail below. Subjects were asked to refrain from alcohol consumption 48 h prior to the second visit and were not allowed to take any over-the-counter medications on the day of the neuroimaging scan. Additionally, subjects were asked to maintain their habitual caffeine usage on the day of the scan to minimize withdrawal effects on brain vasculature.

#### Total Sleep Deprivation (TSD) Session

Subjects returned to the lab for their final visit, which was scheduled 1–4 days following the neuroimaging session (2.4 ± 1.5 days). On the two nights prior to the TSD session, subjects were instructed to go to bed between 22:00 and 23:00 and remain in bed for at least 8 h. Subjects were required to wake no later than 08:00 and received a wake-up call at 07:30 from the research staff on the morning of the TSD session. Compliance was verified by wrist actigraphy. The TSD session began when subjects woke on the day of the visit, and ended at 12:00 the following day, after ~29 h of continuous wakefulness. After awakening on the morning of the TSD session, naps were prohibited until the end of the study. Subjects were also not allowed to consume caffeine for 24 h prior to arriving at the lab. Subjects were also asked to fast, from 13:00 until arrival at the lab later that afternoon (~5.5 h of fasting before arrival).

**Figure 2:**
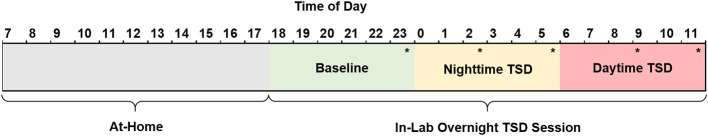
Schematic of the 29 h TSD session. Subjects woke by 08:00, arrived at the sleep laboratory by 18:30, and remained awake until 12:00 the following day at which time they were discharged and allowed to leave the lab. Consumed food was calculated for three periods of the TSD session: baseline (18:00–23:59, green), nighttime TSD (00:00–05:59, yellow), and daytime TSD (06:00–12:00, red). Subjects performed a food rating task (^*^) five times throughout the night. Subjects completed the KSS (not shown) hourly beginning at 19:15. TSD, total sleep deprivation; KSS, Karolinska Sleepiness Scale.

Subjects arrived at the lab by 18:30 and remained awake until they were released at 12:00 the following day (Figure 2). Upon arrival, height and weight measurements were collected and used to calculate body mass index (BMI) following the standard formula [(weight (lbs.)/height (in.)^2^) x 703]. Subjects were allowed to read, play games, and watch TV or DVDs during their free time. Additionally, subjects could access the internet to read news, watch videos, or play games. However, contact with individuals outside of the lab via personal cellphones, social media, chatting, or email was prohibited. Subjects participated in the TSD session in pairs in our controlled laboratory environment, which included a common room and individual testing rooms used for cognitive performance assessments. Light levels were kept at a fixed level and the ambient temperature remained constant for the duration of the study. Further, subjects did not have exposure to natural sunlight while in the lab. A trained research assistant was on staff at all times to administer study procedures and observe behavior throughout the overnight session. Subjects were also monitored with a closed-circuit camera for the duration of the session.

### Neurobehavioral Tasks

Subjects performed a series of computerized neurobehavioral tasks during the baseline neuroimaging session and throughout the overnight TSD session. Here, we will focus on two pre-TSD (i.e., neuroimaging session) fMRI tasks (MSIT and *n-*back task), and one TSD task (Food Rating task). Subjects performed practice sessions of both the MSIT and *n-*back before entering the fMRI scanner.

#### Multi-Source Interference Task

The MSIT (38) was the first of three neurobehavioral tasks performed in the MRI scanner at ~14:20 (± 55 min), following scanner calibration and structural imaging. The MSIT commonly activates the cingulo-frontal parietal network, which is important for attention and monitoring cognitive interference (39). During the task, subjects were shown a series of three numbers (0, 1, 2, or 3). One number (target) was always different from the other two numbers (distractors). Numbers were presented during either control or interference trials. In the control trials, subjects pressed the button that corresponded to the *spatial location* of the target number (i.e., 100, 020, 003), where 0s served as distractors. In the interference trials, subjects pressed the button that corresponded to the *identity* of the target (i.e., different) number (i.e., 221, 233, 322), where identical numbers served as distractors. The MSIT is described in more detail elsewhere (40). Brain activation contrast maps were created for the Interference > Control condition for each individual.

#### *n*-back Task

The *n-*back task was the second of three neurobehavioral tasks performed in the MRI scanner, immediately following the MSIT at ~14:30 (± 55 min). The *n*-back task activates the dorsal lateral prefrontal cortex (DLPFC) and parts of the parietal cortex important for working memory (41). During the task, subjects were presented with a series of letters. Subjects pressed a button to indicate whether or not the current letter on the screen was the pre-specified target letter, the same as the letter displayed one letter earlier in the series (1-back), or the same as the letter displayed two letters back (2-back), depending on the cognitive load of the trial. The *n*-back is described in more detail elsewhere (42). Brain activation contrast maps were created for the 2-back > 0-back condition for each individual.

#### Food Rating Task

During the overnight TSD session, subjects performed a food rating task once every 3 h, beginning at 23:35. During the task, subjects were first asked to rate their current level of hunger on a 7-point Likert scale from 1 (“not at all hungry”) to 7 (“extremely hungry”). Subjects were then shown a total of 70 food and non-food images in a randomized order. Images were either of neutral objects (e.g., flowers, trees, rocks), high-calorie foods (e.g., cheeseburgers, French fries, milkshakes), or low-calorie foods (e.g., fruits, vegetables, whole-grains). Subjects were asked to rate how much they would like to eat each item at that moment on a 7-point Likert scale from 1 (“do not want to eat it”) to 7 (“strongly desire to eat it”). Similar versions of this task have been reported and described elsewhere (20, 43, 44).

#### Karolinska Sleepiness Scale (KSS)

The KSS is a 9-point Likert scale used to measure subjective sleepiness (45). Subjects rated their current level of sleepiness from 1 (“extremely alert”) to 9 (“very sleepy, great effort to keep awake, fighting sleep”). The KSS was administered hourly beginning at 19:15 as part of a larger standardized test battery that is outside the scope of this paper.

### Food

Prior to the start of the TSD session, a trained research assistant prepared identical food baskets for each subject, labeled with the corresponding subject number. For a full list of food items, see the Supplemental Material (Table S1). Subjects recorded all food and drink items they consumed the morning of the TSD session and were required to fast from 13:00 to 18:30 (~5.5 h) prior to entering the laboratory. Following study arrival and intake procedures at 18:30, subjects had *ad libitum* access to 10,900 kcal of food throughout the entire TSD period (see **Table 2** for nutritional breakdown). Subjects discarded food packaging and unwanted leftovers in individualized trash bins labeled with their corresponding subject number. Trained research assistants also observed and recorded food intake throughout the TSD session. Discarded packaging and leftover food items were collected at 6 h intervals (00:00, 06:00, and 12:00). Total calories, calories from fat, grams of fat, grams of carbohydrates, grams of protein, and grams of sugar were documented according to the per serving nutritional values on the food packaging. For items that did not have packaging (apples, bananas) nutritional information was obtained from the U.S. Department of Agriculture Food Composition Database (https://ndb.nal.usda.gov/ndb/). For items partially consumed, values were recorded to the nearest fraction (i.e., ^1^/_4_, ^1^/_3_, ^1^/_2_, ^2^/_3_) of the full nutritional value listed. For analytic purposes, food consumption was broken down into three 6 h periods (Baseline: 18:00–23:59; Nighttime TSD: 00:00–05:59; Daytime TSD: 06:00–12:00) see Figure 2.

### Neuroimaging Methods

Subjects underwent an fMRI scan at the end of the second visit. Neuroimaging scans were collected on a 3.0 Tesla Siemens Tim Trio Scanner (Siemens, Erlangen, Germany) with a 32-channel head coil. First, structural T1-weighted 3D images were collected with a magnetization-prepared rapid gradient-echo (MPRAGE) sequence (repetition time [TR] = 2.1 s; echo time [TE] = 2.3 ms; flip angle (FA) = 12°) over 176 sagittal slices (256 × 256 matrix) with a slice thickness of 1.0 mm (voxel size = 1.0 × 1.0 × 1.0 mm). T2^*^-weighted functional scans were collected over 34 transverse slices (3.5 mm thickness, no gap) using an interleaved sequence (TR = 2.0 s; TE = 30 ms; FA = 90°) with 198 images and 239 images collected per slice for the MSIT and *n*-back, respectively. Data were collected with a 22.4 cm field of view, 64 × 64 acquisition matrix, and a voxel size of 3.5 × 3.5 × 3.5 mm^3^.

### Image Processing

Functional neuroimaging scans were analyzed and processed using Statistical Parametric Mapping software (SPM12; Welcome Department of Cognitive Neurology, London, UK; http://www.fil.ion.ucl.ac.uk/spm/). The raw functional images were first realigned and unwarped. Realigned images were co-registered to each individual's T1-weighted structural image. Subject images were then normalized from the original native space to the 3D space of the Montreal Neurological Institute (MNI) using forward deformation fields. The images were then spatially smoothed with a 6 mm full-width half maximum isotropic Gaussian kernel and resliced to 2 × 2 × 2 mm^3^ voxels using 4th degree B-spline interpolations. Low frequency confounds were removed using a high-pass filter with a 128 s cutoff period. The standard canonical hemodynamic response function for SPM12 was employed, and serial correlation was corrected using a first-order autoregressive model (AR1). The Artifact Detection Tool (ART; http://www.nitrc.org/projects/artifact_detect/) for SPM12 was used to remove motion and spiking artifacts. Scans exceeding 3 standard deviations in mean global intensity, scan-to-scan motion that exceeded 1 mm, and the first scan of each run were regressed out of each 1st level analysis.

### Statistical Analyses

First level analyses were conducted in SPM12 using a general linear model (GLM) to create subject-specific brain activation maps by contrasting the Interference and Control conditions (Interference > Control) on the MSIT and the 2-back and 0-back conditions (2-back > 0-back) on the *n-*back. Contrast images were then entered into separate second level multiple regression models to assess the relationship between well-rested functional activation during the MSIT and *n*-back tasks and caloric and macronutrient consumption during the three periods of the TSD session. Gender, BMI, and caloric/macronutrient intake during baseline (18:00–00:00) were included as covariates for nighttime TSD and daytime TSD analyses.

Whole brain-analyses were initially height thresholded at *p* < 0.001 (uncorrected). Cluster-level statistics were corrected for family-wise error (FWE) at *p* < 0.05. For *post-hoc* analyses, the first eigenvariates for the significant clusters were extracted from SPM12 for regression and plotting purposes in SAS (v9.4). Additionally, Pearson's partial correlations, controlling for BMI and gender, were used to compare caloric and macronutrient intake to baseline and TSD subjective sleepiness levels. Simple linear regression models were used to individually assess the relationship between individual caloric intake and macronutrient intake and subjective hunger food desire ratings and KSS score as a function of time awake. Additionally, linear regression was used to assess the relationship between BMI and caloric/macronutrient and gender and caloric/macronutrient intake during each TSD period.

## Results

### Subject Characteristics

Subject characteristics are summarized in Table 1.

**Table 1:** Subject characteristic.

**Characteristic**	**Mean (±*SD*)**
Age (y)	25.4 ± 5.6
Height (m)	1.7 ± 0.1
Weight (kg)	72.7 ± 12.9
BMI (kg/m^2^)^a^	23.9 ± 3.7
Normal weight (*n* = 30)	21.8 ± 1.9
Overweight (*n* = 12)	27.3 ± 1.3
Obese (*n* = 3)	31.8 ± 1.6
Time in bed (min)^b^	501.7 ± 77.4
Sleep duration (min)^b^	448.7 ± 72.7
Bedtime (hh:mm ± min)^b^	22:56 ± 68.7
Wake time (hh:mm ± min)^b^	07:17 ± 61.4

a *BMI categories are defined as follows: Normal: 18.5–24.9 kg/m^2^; Overweight: 25.0–29.9 kg/m^2^; Obese: >30.0 kg/m^2^*.

b *Time in bed, sleep duration, and bed and wake times were derived from wrist actigraphy for the one night prior to the TSD period*.

### Caloric and Macronutrient Intake

During the at-home portion of the study on the first day of sleep deprivation (~11 h total), subjects consumed an average of 1135.2 ± 414.9 total calories and 379.9 ± 232.1 calories from fat prior to entering the laboratory. They consumed 158.2 ± 169.8 g of carbohydrates, 52.2 ± 27.0 g of sugar, 117.1 ± 1.0 g of fat, and 45.6 ± 23.3 g of protein. Throughout the in-laboratory portion of the TSD session (17 h total), subjects consumed an average of 2,503.4 ± 754.0 total calories of the 10,900 calories available. On average, they consumed 769.4 ± 302.6 calories from fat, 380.7 ± 124.6 g of carbohydrates, 187.9 ±75.5 g of sugar, 85.5 ± 33.6 g of fat, and 61.2 ± 18.9 g of protein. Total caloric and macronutrient intake for the overnight session exceeded the recommended daily intake (RDI) values for an entire day based on a 2,000-calorie diet (Table 2).

**Table 2:** Caloric and macronutrient intake across the in-laboratory sleep deprivation session.

		**At-Home****^b^**		**Baseline**		**Nighttime TSD**		**Daytime TSD**		**Total TSD**		
		**08:00–13:00**		**18:00–23:59**		**00:00–05:59**		**06:00–11:59**				
	**Available**^a^****	**Mean**	***SD***	**Mean**	***SD***	**Mean**	***SD***	**Mean**	***SD***	**Mean**	***SD***	**% RDI****^c^**
Total calories (kcal)	10,900	1135.2	414.9	1175.2	468.1	780.9	419.0	547.4	226.9	2503.4	754.0	125.2
Calories from fat (kcal)	2,700	379.9	232.1	414.8	207.1	246.1	162.9	108.6	91.9	769.4	302.6	109.9
Carbohydrates (g)	1,400	158.2	69.6	163.5	77.2	117.1	64.7	100.1	40.0	380.7	124.6	138.4
Sugar (g)	600	52.2	27.0	75.5	44.5	55.0	36.9	57.4	24.4	187.9	75.5	375.7
Fat (g)	300	117.1	169.8	46.2	23.1	27.3	18.1	12.0	10.2	85.5	33.6	109.6
Protein (g)	200	45.6	23.3	29.2	11.7	19.2	12.2	12.8	8.5	61.2	18.9	122.4

a *Values are rounded to the nearest 100. Available values refer to in-lab portion only*.

b *Self-reported food diaries were used to calculate nutritional information. Subjects were required to fast from 13:00–18:30 prior to arriving at the lab*.

c *Recommended Daily Intake (RDI) as set by the United States Food and Drug Administration*.

Body mass index (BMI) did not predict total caloric intake during any of the three TSD periods (baseline: [*F*_(1, 43)_ = 2.80, *p* = 0.10, *R*^2^ = 0.06]; nighttime TSD: [*F*_(1, 43)_ = 2.56, *p* = 0.12, *R*^2^ = 0.06]; daytime TSD: [*F*_(1, 43)_ = 1.96, *p* = 0.17, *R*^2^ = 0.04]). Likewise, BMI did not predict total calories from fat, grams of fat, or grams of carbohydrates for any of the TSD periods [*F*_(1, 43)_ < 3.48, *p* > 0.07]. However, higher BMI was significantly associated with more grams of sugar consumed during baseline [*F*_(1, 43)_ = 5.66, *p*=0.02], but not during nighttime or daytime TSD. Higher BMI was also significantly associated with more grams of protein consumed during nighttime TSD [*F*_(1, 43)_ = 4.63, *p* = 0.04], but not during baseline or daytime TSD. Gender was not a significant predictor of total caloric intake during any of the three TSD periods (baseline: [*F*_(1, 43)_ = 1.69, *p* = 0.20]; nighttime TSD: [*F*_(1, 43)_ = 0.0, *p* = 0.97]; daytime TSD: [*F*_(1, 43)_ = 0.09, *p* = 0.76]). Gender did not predict total calories from fat, grams of fat, grams of carbohydrates, or grams of protein consumed during any of the TSD periods [*F*_(1, 43)_ < 3.13, *p* > 0.08]. However, males consumed significantly more sugar during baseline [*F*_(1, 43_ = 4.81, *p* = 0.03] compared to females. These differences were not apparent during nighttime or daytime TSD. Full statistical results can be found in the Supplemental Material (Table S2).

### Neural Correlates of Caloric and Macronutrient Intake

**Figure 3:**
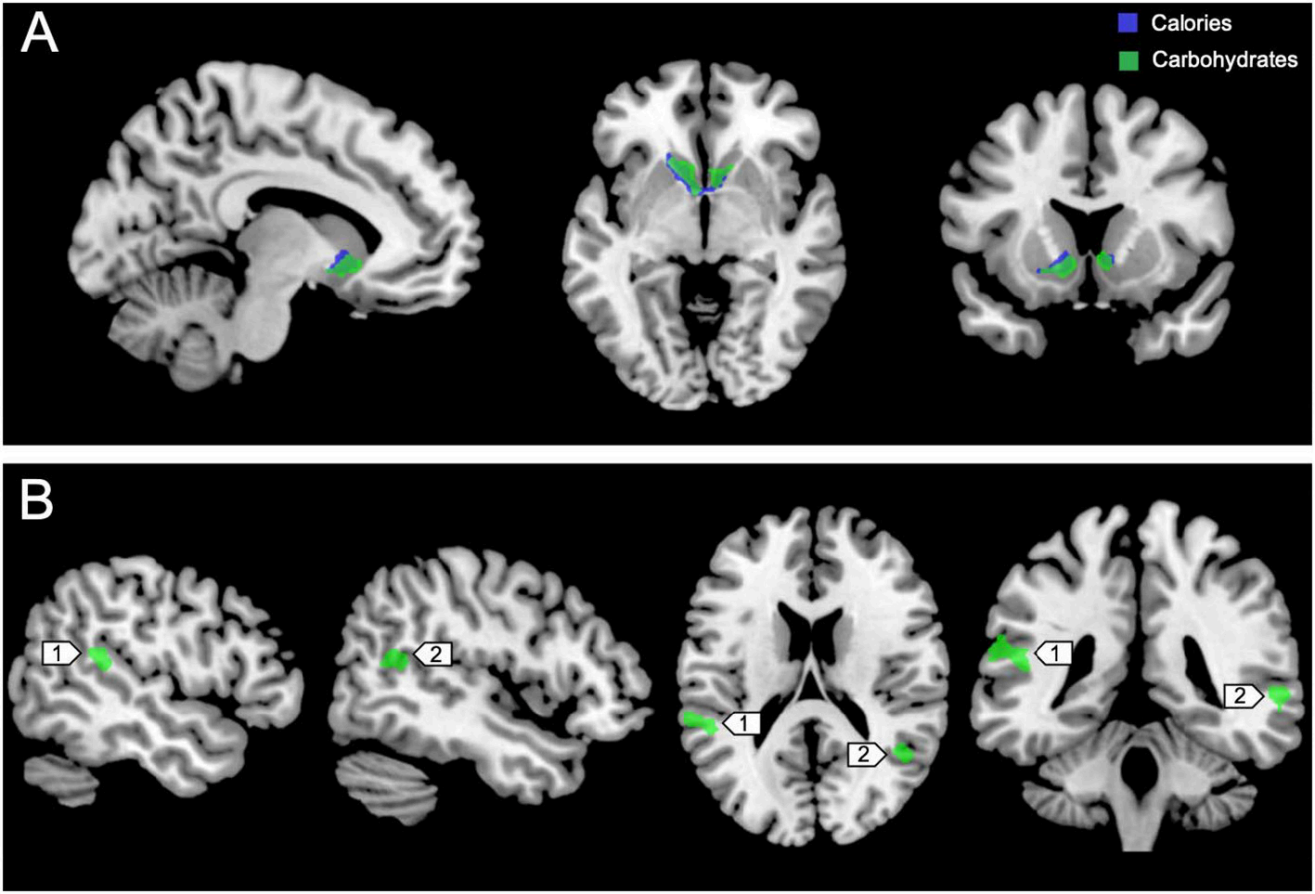
Whole-brain analyses (*p* < 0.001, cluster corrected; *p* < 0.05, FWE) of MSIT activation during the Interference > Control condition. **(A)** Sagittal, axial, and coronal views of significant activation in the nucleus accumbens (MNI: −10, 10, −8) as it correlates with total calories (blue) and total carbohydrates (green) consumed during the last 6 h of sleep deprivation (i.e., 06:00–12:00). **(B)** Significant activation in the left superior temporal gyrus (1; MNI:−64, −36, 18) and right middle temporal gyrus (2; MNI: 64, −46, 4) as it correlates with total carbohydrates (green) consumed during the last 6 h of sleep deprivation. FWE, Family-wise error; MSIT, Multi-Source Interference Task; MNI, Montreal Neurological Institute.

**Figure 4:**
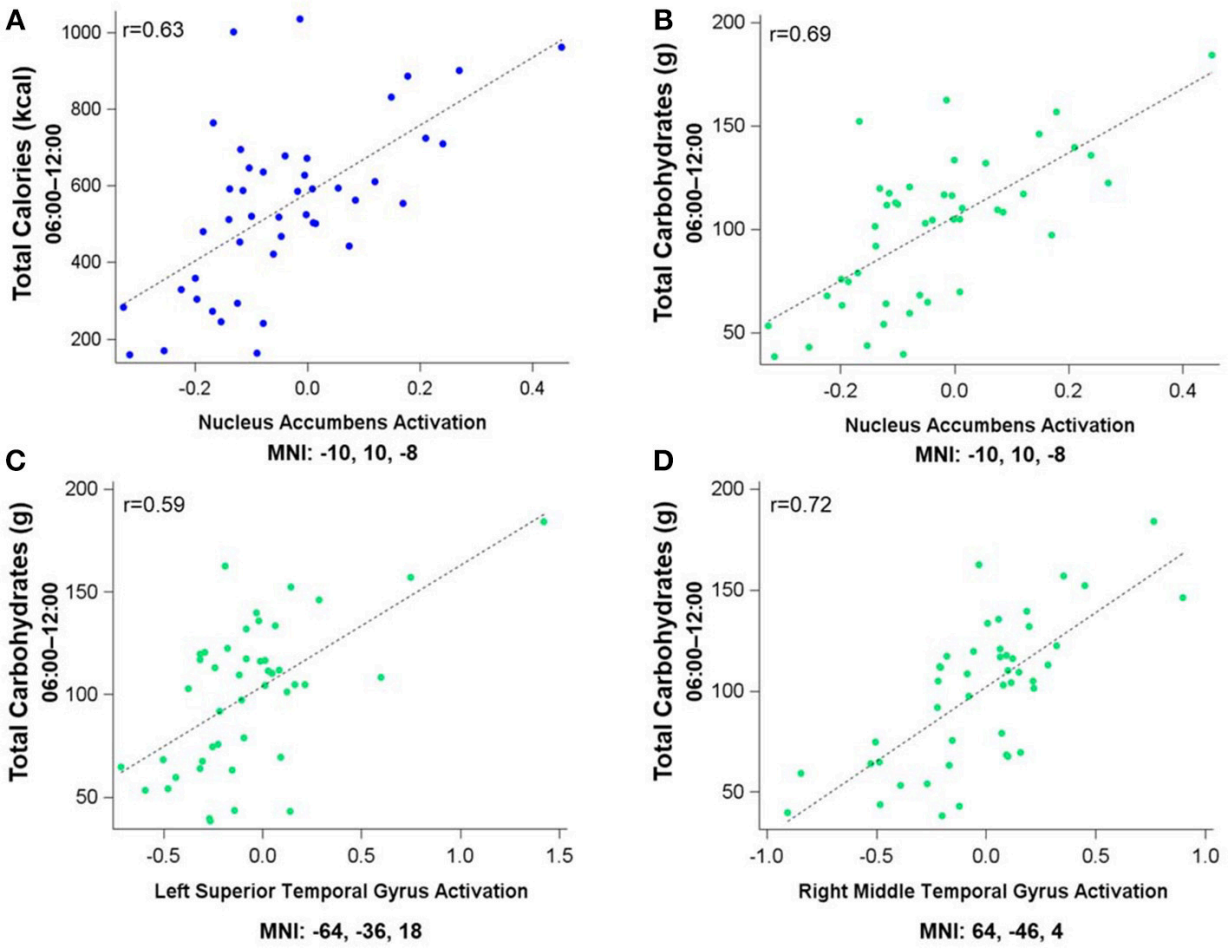
Residualized eigenvariates for the neural activation in the left nucleus accumbens during the MSIT Interference > Control condition as it relates to **(A)** total calories and **(B)** total carbohydrates consumed during the last 6 h of sleep deprivation and **(C,D)** activation in the temporal gyri as it relates to total carbohydrates consumed during the last 6 h of sleep deprivation.

Figure 3 shows the clusters (FWE corrected) with significant correlations between MSIT (Interference > Control) performance and total caloric (blue) or carbohydrate (green) intake. While no regions of the brain were associated with calorie consumption during the baseline and overnight periods, we observed a significant correlation between activation within the ventral striatum and calories consumed (*p* = 0.024) in the next-day period between 06:00 and 12:00 during TSD (Figure 3A). This pattern was accounted for primarily by a significant correlation in the ventral striatum for grams of carbohydrates consumed (*p* = 0.016) during the same timeframe (Figure 3A). In addition, we observed significant correlations in the right middle temporal gyrus (*p* < 0.001) and the left superior temporal gyrus (*p*=0.038) for grams of carbohydrates consumed (Figure 3B). Activation in the reward circuitry, specifically the NAc, while performing the MSIT under well-rested conditions predicted both total calories consumed and total carbohydrates consumed during hours 23–29 of continuous wakefulness (i.e., the last 6 h of the TSD session; Figure 3A). Similarly, activation in areas associated with viewing unhealthy food (30) (middle and superior temporal gyri) predicted increased carbohydrate consumption (Figure 3B). Higher activation in each region was associated with increased caloric and carbohydrate consumption (Figure 4). However, activation in these regions did not predict consumption of total calories from fat, grams of sugar, grams of fat, or grams of protein. Neuroimaging results are summarized in Table 3.

**Table 3:** Cluster maxima for whole-brain multiple regression analyses of the MSIT Interference > Control condition and *n-*back 2-back > 0-back condition as it pertains to caloric and carbohydrate consumption.

	**Anatomical region**	**Hemisphere**	**Cluster size**	***T*_**40**_**	**Cluster *p*^a^**	**x**	**y**	**z**
**MSIT**								
*Calories*	Nucleus accumbens	L	181	5.15	0.024	−10	10	−8
*Carbohydrates*	Nucleus accumbens	L	196	5.97	0.016	−10	10	−8
	Middle temporal gyrus	R	423	6.39	<0.001	64	−46	4
	Superior temporal gyrus	L	158	4.47	0.038	−64	−36	18
***n*****-back**								
*Calories*	Nucleus accumbens	L	226	4.41	0.026	−4	18	2
*Carbohydrates*	Nucleus accumbens	L	266	4.46	0.013	−4	18	2

a *Cluster level analyses were family-wise error corrected at p < 0.05, with whole-brain analyses thresholded at p < 0.001. MSIT, Multi-Source Interference Task*.

**Figure 5:**
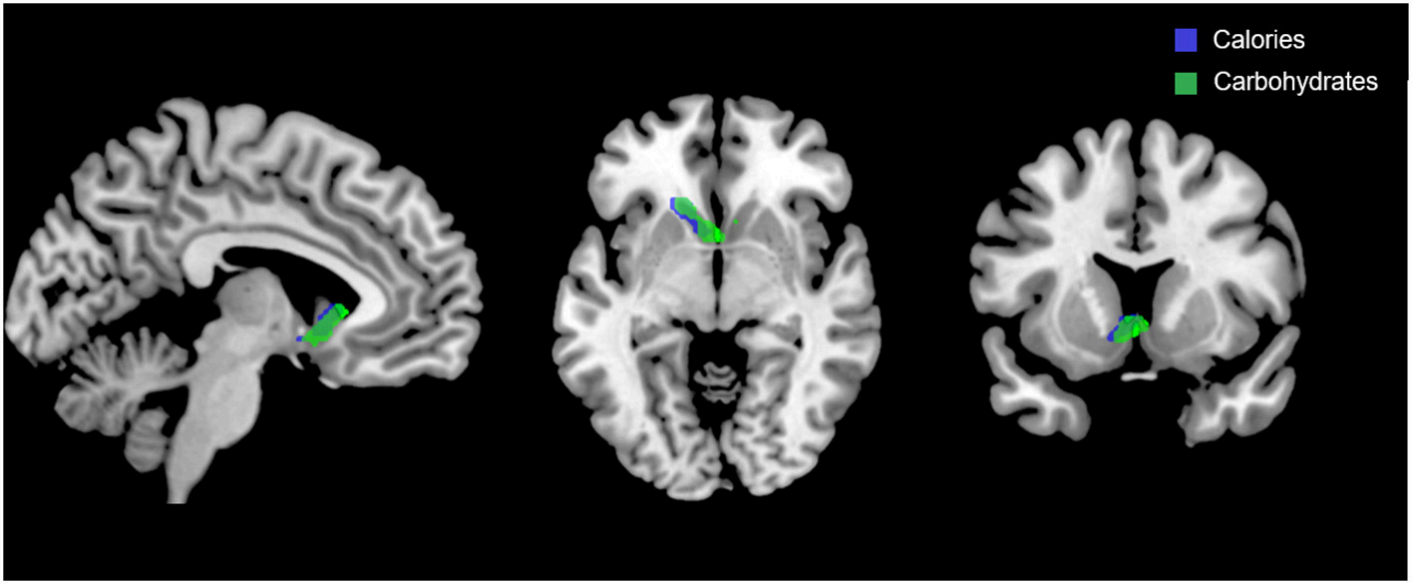
Whole-brain analyses (*p* < 0.001, cluster corrected; *p* < 0.05, FWE) of *n*-back activation during the 2-back > 0-back condition. Sagittal, axial, and coronal views of significant activation in the nucleus accumbens (MNI:−4, 18, 2) as it correlates with total calories (blue) and total carbohydrates (green) consumed during the last 6 h of sleep deprivation (i.e., 06:00–12:00). FWE, Family-wise error; MNI, Montreal Neurological Institute.

**Figure 6:**
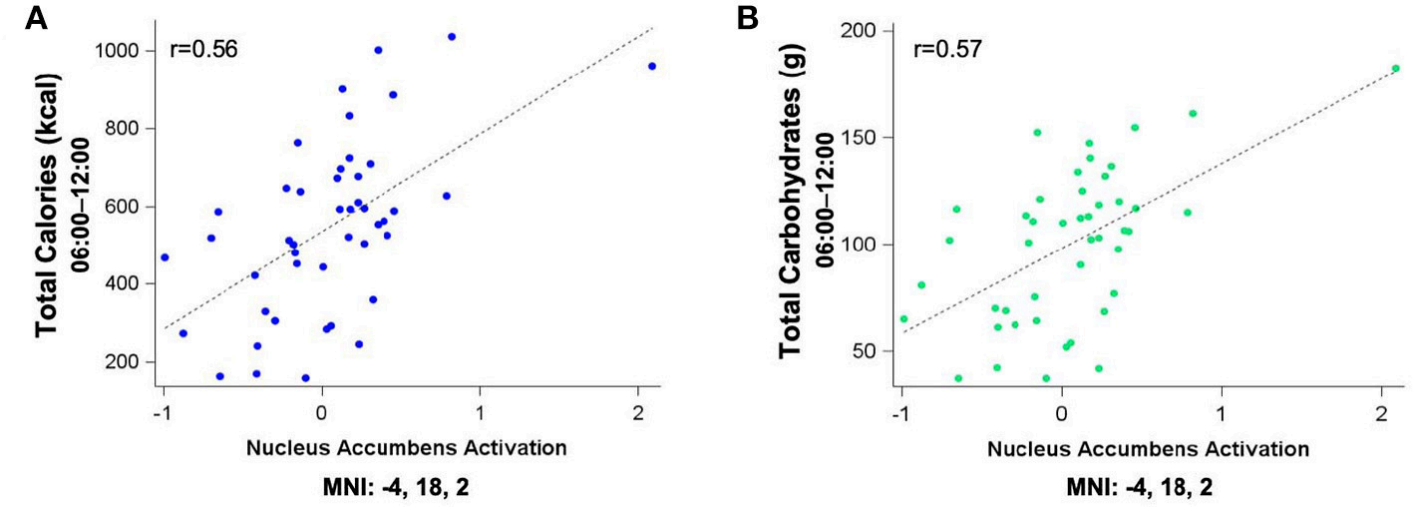
Residualized eigenvariates for the neural activation in **(A)** the left nucleus accumbens during the *n-*back 2-back > 0-back condition as it relates to total calories (blue) and **(B)** total carbohydrates (green) consumed during the last 6 h of sleep deprivation.

Figure 5 shows the clusters (FWE corrected) with significant correlations between *n-*back performance (2-back > 0-back) and total caloric (blue) and carbohydrate (green) intake. Critically, there was no association between brain activation and calorie consumption at baseline or during the night. However, similar to the previous findings for the MSIT, we observed a significant correlation between activation of the ventral striatum during the *n*-back and subsequent calories consumed (*p* = 0.026) during the morning between 06:00 and 12:00 during TSD (Figure 5). Likewise, we observed a significant correlation in the ventral striatum for grams of carbohydrates consumed (*p* = 0.013) in the same timeframe (Figure 5). Similar to the MSIT, activation in the NAc while performing the *n*-back under well-rested conditions predicted both total caloric intake and grams of carbohydrates consumed during the last 6 h of TSD. Higher activation in this region was associated with higher caloric and carbohydrate intake (Figure 6). However, like the MSIT, activation did not predict consumption of total calories from fat, grams of sugar, grams of fat, or grams of protein. Neuroimaging results are summarized in Table 3.

### Subjective Sleepiness (KSS) Ratings

Subjective ratings of sleepiness were low during the baseline period (3.73 ± 0.73) and steadily increased across the nighttime (5.78 ± 2.01) and daytime TSD hours (6.41 ± 2.00) as expected with extended wakefulness. KSS scores during the baseline, nighttime, and daytime TSD periods were not significantly correlated with total caloric (*r* < 0.10, *p* > 0.53) or carbohydrate (*r* < 0.16, *p* > 0.30) intake (Figure S1). Full statistical results are reported in the Supplemental Material (Table S3).

### Subjective Hunger and Food Desire Ratings

**Figure 7:**
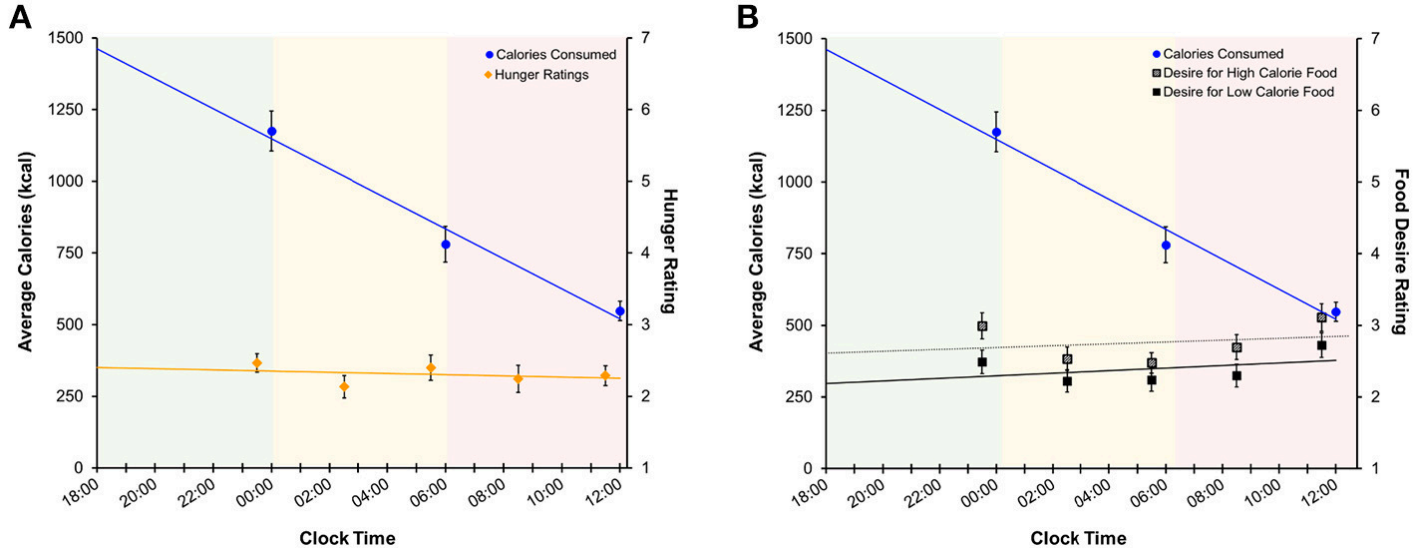
Regression models of mean (±SE) **(A)** calories consumed (left axis) and subjective hunger ratings (right axis) and **(B)** calories consumed and food desire ratings across the sleep deprivation period. Shaded areas represent the three TSD periods. Green: baseline; yellow: nighttime TSD; red: daytime TSD. TSD, total sleep deprivation.

Average subjective hunger ratings were low during both the nighttime (2.34 ± 0.89) and daytime TSD (2.26 ± 1.17) periods. Figure 7 shows subjective hunger ratings and desire ratings for high- and low-calorie foods across the TSD period in relation to total caloric intake. Regression analyses indicated that average hunger ratings did not change across the sleep deprivation session [*F*_(1, 223)_ = 0.12, *p* = 0.73, *R*^2^ < 0.001]. Likewise, there was no change in desire for high-calorie [*F*_(1, 223)_ = 2.32, *p* = 0.13, *R*^2^ = 0.01] or low-calorie [*F*_(1, 223)_ = 3.03, *p* = 0.08, *R*^2^ = 0.01] foods across time. However, total caloric and carbohydrate intake did change across the sleep deprivation period (calories: [*F*_(1, 133)_ = 59.49, *p* < 0.001, *R*^2^ = 0.31]; carbohydrates: [*F*_(1, 133)_ = 23.11, *p* < 0.001, *R*^2^ = 0.15]), generally declining with longer time awake. Hunger ratings were stable for the duration of the overnight session.

## Discussion

This study investigated whether pre-TSD neural activation in reward-related brain regions was associated with total caloric and macronutrient intake during one night of sleep deprivation. On average, subjects consumed 2,500 kcal throughout the 17 h in-lab portion of the TSD session, exceeding the recommended total daily value of 2,000 kcal (46). The observed caloric intake during extended wakefulness was similar to other sleep restriction (18) and sleep deprivation studies (28). Moreover, we found that functional activation in the bilateral NAc (most prominently on the left) during two independent cognitively demanding tasks (i.e., MSIT and *n-*back) pre-TSD (Figures 3A, 5) was significantly associated with total caloric (Figures 4A, 6A) and carbohydrate (Figures 4B, 6B) intake during the last 6 h of TSD (06:00–12:00). In addition, while not predicted, we found that activation within the middle and superior temporal gyri (Figure 3B) also correlated with total carbohydrate consumption during the same timeframe (Figures 4C,D). Baseline activation was not associated with calories from fat, grams of fat, grams of protein, or grams of sugar consumed during any portion of the overnight TSD session. To our knowledge, this is the first study to demonstrate that individual differences in baseline activation of the ventral striatum are potentially *predictive* of eating behaviors several days later during one night of TSD.

Our findings suggest that individuals with greater baseline responsiveness within the reward system (i.e., NAc) when well-rested may be most vulnerable to overeating during subsequent sleep deprivation. As outlined in greater detail in the sections that follow, we suggest that when considered in light of the well-known decreases in prefrontal inhibitory control that occur during sleep deprivation, those individuals with the greatest sustained NAc reward responses may be most prone to overconsuming calories when lacking sleep. Excess reward responsiveness in conjunction with sleep loss-induced deficits in prefrontal inhibitory control is likely to represent a problematic combination when one encounters attractive high-calorie foods.

Our findings complement evidence from previous studies assessing the neural responses to food cues and subsequent food intake (47) or weight gain (48) during rested conditions and studies assessing neural responses to food stimuli during sleep restriction or TSD (26, 27, 29, 31). Studies using fMRI have shown increased neural reactivity in reward-related brain regions, specifically in the NAc, in response to food images during both rested (47, 48) and sleep-restricted conditions (27, 31). St. Onge et al. (31) found that when sleep was restricted to 4 h per night for six nights, subjects showed increased neural activity in reward-related regions, including the NAc and the superior temporal gyri, when viewing images of food vs. images of non-food items (31). In a follow-up study, they found that viewing unhealthy foods during sleep restriction increased neural activation in several cortical regions, including the middle and superior temporal gyri (30). Further, the temporal gyri have been implicated in the perception and cognitive processing of emotional stimuli (49), including increased neural responses to food stimuli during a satiated state (50). Individuals diagnosed with anorexia nervosa, compared to controls, do not show such elevated responses, suggesting that altered activation patterns in these regions may be related to increased responsiveness to the pleasurable characteristics of food, and therefore may contribute to the control of food intake (50). We found that high pre-TSD activation in these cortical areas is strongly associated with carbohydrate consumption during TSD. Additionally, a recent study by Demos et al. (27) found that when sleep was restricted to 6 h per night for four nights, neural activity increased in the left and right NAc when viewing images of food (27). In addition to findings in studies of sleep restriction, studies in well-rested individuals have shown that increased reactivity of the NAc to food stimuli is associated with both weight gain (48) and increased snacking behavior (47). Taken together, these studies demonstrate that individual differences in responsivity of the fronto-striatal-midbrain reward circuitry contribute to increased hedonic food consumption during sleep loss. While we did not measure fMRI activation *during* sleep deprivation, prior evidence suggests that sleep loss does in fact alter activation patterns of the reward system in response to food stimuli (26, 27, 29, 31). Therefore, it is plausible that individuals with increased striatal activity prior to sleep loss, as shown here, may be more susceptible to TSD-induced perturbations to the underlying neurocircuitry associated with reward-driven behaviors, thus contributing to the tendency to overeat.

Imaging studies in obese individuals suggest that there is a discrepancy between the enhanced sensitivity of an expected reward (i.e., seeing food) and a decreased sensitivity to the gratifying effects of the reward (i.e., eating food). That is, there is an increased propensity to overeat because the reward expectation is never met (22). We propose that a similar phenomenon may be occurring during TSD, especially among those individuals with elevated baseline reward sensitivity. The data here suggest that individuals with higher baseline neural responsiveness within the NAc have a “reward anticipation reserve,” meaning that they are more likely to expect a reward from their actions (25), but the reward expectations may not be fully met.

While not directly assessed in the present study, we speculate dopamine^1^ may play a mechanistic role in the present findings. Dopamine is a key neurotransmitter in reward circuits and underlies the pleasurable properties of food. Overweight and obese individuals show signs of disrupted reward processing within the striatum, including altered neural activity and dopamine release, as well as decreased dopamine D_2_ receptor availability (22, 52). Interestingly, sleep loss impairs dopamine signaling in a similar manner, such that dopamine D_2_ receptors are downregulated, (53, 54) and neural activation in the ventral striatum is increased, (27, 29–31) both of which are similar to findings in overweight and obese individuals. Due to the downregulation of D_2_ receptors in the striatum, it is probable that dopamine is unable to bind effectively to the limited number of D_2_ receptors that are available. Therefore, dopamine signals that indicate a reward expectation have been met are potentially disrupted. The effect that sleep loss has on dopamine function within the striatum may be amplified in individuals with elevated baseline reward sensitivity, making these individuals more vulnerable to overeating during sleep loss. Alternatively, the reduction of dopamine D_2_ receptor availability may push the system into a D_1_ receptor dominated state, thereby promoting reward-driven behavior, such as eating (55).

Dopamine signaling within the striatum is also linked to the receptor activity of a well-characterized neural substrate, adenosine. Adenosine accumulates with extended wakefulness and has been implicated in homeostatic sleep regulation, as it inhibits neural activity in wake-promoting regions of the brain (56). Within the striatum, dopamine D_2_ receptors are co-localized with adenosine A_2A_ receptors and functionally interact in an antagonistic manner (57). That is, the binding of adenosine to the A_2A_ receptors inhibits the actions of the dopamine D_2_ receptors, thus impairing downstream dopamine-related neurotransmission. The inhibition of D_2_ receptor activity by adenosine, in combination with the downregulation of D_2_ receptors during sleep loss, may also contribute to overeating by further disrupting normal reward signaling pathways.

In addition to increased reward sensitivity and disrupted dopamine signaling in the basal ganglia, there is evidence from positron emission topography (PET) imaging that one night of TSD reduces glucose metabolism within the PFC, including the vmPFC (24). The vmPFC is considered an inhibitory emotional control region, and dysfunction may contribute to a loss of inhibitory control over subcortical reward regions, such as the NAc. Loss of inhibitory control over emotional responses can lead to increased risky behavior and impulsivity, potentially increasing the tendency to overeat (58–61). Risky decisions during sleep loss are associated with increased NAc activation, (25) similar to how the brain responds when viewing images of food when sleep restricted (27, 31). This increase in neural activation during risky decision making presumably results in an elevated expectation of reward (25). Simultaneously, neural activation within the OFC is reduced, suggesting an attenuated ability to learn from any negative consequences from the risky behavior, (25) such as weight gain associated with increased caloric intake. Additionally, reduced PFC glucose metabolism is correlated with reduced D_2_ receptor availability in obese individuals, further implicating impaired inhibitory control in overeating (52). Taken together, these earlier studies suggest that in combination with individual differences in baseline NAc activation, sleep loss may amplify the inability to inhibit impulsive eating behavior and increase the expectation of reward from eating, ultimately leading to over consumption of high calorie foods and carbohydrates.

Similar to findings in previous studies (27, 29, 47) we found no association between hunger ratings and food consumption during TSD (Figure 7A and Figure S2A), as hunger ratings remained stable throughout each period of the TSD session. Desire for high- and low-calorie foods also remained stable across the night (Figure 7B and Figure S2B). While both hunger and food desire ratings remained stable, it should be noted that overall caloric (Figure 7) and carbohydrate (Figure S2) intake decreased as a function of time awake. However, subjects consumed roughly 60% of the recommended RDI within the first 6 h of the study (Table 2), suggesting that the decline in consumption is a result of satiation. It is important to note that subjects still consumed a significant number of calories during the nighttime and daytime TSD periods, and the findings for the final morning period were statistically controlled for calorie consumption in the prior periods. Further, we did not find evidence that subjective sleepiness is correlated with increased caloric and carbohydrate intake (Figure S1). Taken together, our results suggest that: (1) hunger or desire for food was not the primary driving force for the excessive food consumption demonstrated here; and (2) subjects were not overeating due to increased sleepiness or as a means to stay awake. These findings offer further support of the mechanistic theory that impaired functioning within the reward circuitry contributes to heightened hedonic motivations for food during TSD.

The present study demonstrates a strong association between pre-TSD reward sensitivity and the tendency to overeat during sleep loss. However, several limitations should be addressed. First, neuroimaging scans were not collected during the TSD session, which limits our ability to understand the dynamic changes in brain activation patterns as they relate to caloric and macronutrient intake, hunger, desire for food, and sleepiness. Further, it remains unknown whether or not the findings presented are unique to TSD, or whether the neural activation and food consumption patterns are also observed under well-rested control conditions. However, it is important to note that we only found a significant relationship between pre-TSD activation and food consumption during hours 23–29 of wakefulness, and not during the first 12 h of the in-lab TSD session, suggesting that the effects are only present following a sufficient amount of sleep loss. Future studies should assess the relationship between brain activation patterns and food consumption in well-rested and TSD groups. Second, although subjects provided a self-report log of foods consumed prior to arriving at the laboratory, it is impossible to completely verify compliance with the 5 h fasting period before the in-lab portion of the study. Third, it should be noted that our study population consisted of young, healthy adults, and we do not know how our findings are generalizable to other populations. Additionally, increased caloric and macronutrient intake during sleep loss, and its relation to obesity, is a multifaceted problem. Eating behaviors may be modified by a number of physiological and environmental factors including changes in appetite and satiety hormones, meal timing, gender, BMI, genetics, and lifestyle (6, 62). Our BMI range was not restricted, and included individuals classified as either normal, overweight, or obese (Table 1). While we showed no association between BMI and caloric/carbohydrate consumption, this factor should not be overlooked in future studies as some evidence suggests that brain responses to food images differs among lean and obese individuals (63, 64). In addition, we showed no gender differences in total caloric or carbohydrate intake, only differences in baseline sugar consumption. Due to the limited scope of the paper, we did not assess lifestyle, hormones, or genetic markers as possible additional factors that may modulate some of the hedonic pathways discussed here.

Overall, we demonstrated that pre-TSD activation within the ventral striatum, as well as the middle and superior temporal gyri, is associated with eating behaviors during a single night of sleep loss. Individuals with elevated neural activity in these regions consumed significantly more calories and carbohydrates after a night of sleep deprivation. These findings suggest that there are large individual differences in baseline functioning within hedonic reward pathways and sleep loss further disrupts functioning in these pathways. Elevated reward sensitivity appears to impact eating behaviors during sleep loss and may be a major contributor to the etiology of sleep loss related obesity.

## Author Contributions

BS conducted the MRI data processing and statistical analyses, and drafted the initial manuscript. AR assisted with manuscript revisions. WK designed the study, secured funding, collected the data, assisted with data interpretation, and critique, as well as contributed to manuscript review and revisions.

### Conflict of Interest Statement

The authors declare that the research was conducted in the absence of any commercial or financial relationships that could be construed as a potential conflict of interest.

## Footnotes

^1^Dopamine exerts its effects on reward circuits by binding to either D_1_ or D_2_ receptor subtypes located throughout the brain, including the fronto-striatal reward pathways. It should be noted that these subtypes have different functional implications that are beyond the scope of this paper (51).

## Abbreviations

ACC: Anterior Cingulate Cortex
ART: Artifact Detection Tool
BMI: Body Mass Index
DLPFC: Dorsal Lateral Prefrontal Cortex
GLM: General Linear Model
fMRI: Functional Magnetic Resonance Imaging
FWE: Family-Wise Error
kcal: Kilocalories
KSS: Karolinksa Sleepiness Scale
MINI: Mini International Neuropsychiatric Interview
MNI: Montreal Neurological Institute
MSIT: Multi-Source Interference Task
NAc: Nucleus Accumbens
OFC: Orbital Frontal Cortex
PET: Positron Emission Tomography
PFC: Prefrontal Cortex
RDI: Recommended Daily Intake
SPM: Statistical Parametric Mapping
TE: Echo Time
TR: Repetition Time
TSD: Total Sleep Deprivation
vmPFC: Ventromedial Prefrontal Cortex.
