# Supplementary material for: Rested-Baseline Responsivity of the Ventral Striatum Is Associated With Caloric and Macronutrient Intake During One Night of Sleep Deprivation
Source: Front Psychiatry. 2019 Jan 17;9:749. doi: 10.3389/fpsyt.2018.00749 (PMC6344438; doi:10.3389/fpsyt.2018.00749)
Supplement: Supplementary file 1 [file Data_Sheet_1.PDF]

Supplementary Material

Rested-baseline Responsivity of the Ventral Striatum is Associated with Caloric and Macronutrient Intake during One Night of Sleep Deprivation

Brieann C. Satterfield, PhD[a], Adam C. Raikes, PhD[a], and William D.S. Killgore, PhD[a,b*]

[a]Social, Cognitive, and Affective Neuroscience Laboratory, Department of Psychiatry, College of Medicine, University of Arizona, Tucson, AZ, USA
[b]Department of Psychiatry, McLean Hospital, Harvard Medical School, Belmont, MA, USA

*** Correspondence:** Dr. William DS Killgore, killgore@psychiatry.arizona.edu

Table S1. List of food items provided to each subject.

Food Item[a]	Calories[b] (kcal)	Calories From Fat (kcal)	Fat (g)	Carbs (g)	Protein (g)	Sugar (g)
Beverage – 1% Milk (x2)	380	40	4.5	22	15	21
Beverage – Ginger Ale (x2)	240	0	0	34	0	32
Beverage – Lemon-Lime (x2)	280	0	0	38	0	38
Beverage – Lemon-Lime Zero Sugar (x2)	0	0	0	0	0	0
Beverage – Orange (x2)	340	0	0	39	3	34
Beverage – Orange (x2)	320	0	0	43	0	43
Beverage – Root Beer (x2)	320	0	0	43	0	43
Beverage – V8 Vegetable (x2)	60	0	0	7	1	5
Box Meal – Beef Stew	300	60	6	38	22	10
Box Meal – Chicken & Mashed Potatoes	210	30	3.5	26	19	0
Box Meal – Cup of Noodles (Chicken)	290	110	12	39	7	2
Box Meal – Cup of Noodles (Vegetable)	290	100	11	38	7	2
Box Meal – Easy Mac Cup	220	25	3	41	6	6
Box Meal – Spinach & Cheese Ravioli	230	45	5	37	8	7
Box Meal – Turkey & Dressing	290	80	9	31	20	4
Breakfast – Muffin Bites (Blueberry)	180	70	8	25	2	14
Breakfast – Pop-Tarts (Strawberry)	400	90	10	76	4	32
Breakfast – Yogurt (Strawberry)	170	15	1.5	33	5	26
Candy – Skittles (x2)	127	5	0.7	14	0	11
Candy – Sour Patch Kids (x2)	110	0	0	13.5	0	9.5
Candy – Starburst (x2)	80	7.5	0.9	8.3	0	5.5
Candy – Swedish Fish (x2)	110	0	0	13.5	0	11
Cereal – Brown Sugar Oatmeal	200	25	2.5	42	5	18
Cereal – Cheerios	100	15	1.5	19	3	1
Cereal – Cinnamon Toast Crunch	160	35	4	31	2	12
Cereal – Frosted Cheerios	120	10	1.5	25	2	10
Cereal – Golden Grahams	140	10	1.5	30	2	12
Cereal – Honey Nut Cheerios	120	15	1.5	25	3	11
Cereal – Lucky Charms	130	10	1.5	27	3	13
Cereal – Trix	120	15	1.5	27	1	10
Chips – Barbeque	160	90	10	15	2	2
Chips – Cheetos	150	90	10	13	2	1
Chips – Chili Cheese Fritos	160	90	10	15	2	1
Chips – Cool Ranch Doritos	150	70	8	18	2	0.5
Chips – Sour Cream and Onion	160	90	10	15	2	0.5
Chips – Spicy Nacho Doritos	140	70	8	16	2	0
Cookies – Fig Newton	200	40	4	40	1	24
Cookies – Oatmeal Raisin	170	60	6	27	2	12
Crackers – Cheez-It	180	80	9	20	4	0
Crackers – Goldfish	130	40	4.5	19	3	0.5
Crackers – Peanut Butter Sandwich	190	80	9	23	4	5
Crackers – Ritz (x2)	440	110	12	26	2	4
Fruit – Apple (x2)	180	0	0	24	0	15
Fruit – Banana (x2)	210	4	0.4	27	1.3	14.4
Fruit – Diced Peaches in Light Syrup	70	0	0	17	0	16
Fruit – Natural Applesauce	50	0	0	13	0	11
Salad – Orchard Harvest Salad	500	340	38	26	12	18
Salad[c] – Lemon Chicken Salad (n=10 subjects)	440	220	25	42	15	11
Salad[c] – Waldorf Chicken Salad (n=35 subjects)	340	160	18	38	7	12

Snack – Oat and Honey Granola Bar	190	60	6	29	4	12
Snack – Blueberry Nutrigrain Bar	120	30	3	24	2	12
Snack – Fruit Snacks	80	0	0	19	0	10
Snack – Hummus and Pretzels	260	170	19	16	7	1
Snack – Jell-O (Sugar Free Strawberry (x2))	20	0	0	0	1	0
Snack – Natural Almonds	100	80	9	4	4	1
Snack – Pudding (x2)	120	10	1	12	0.5	0
Snack – Slim Jim (x2)	80	30	3	1	2	0
Snack – String Cheese (x2)	140	50	6	0	5	0
Vegetable – Baby Carrots (x2)	50	0	0	6	1	4

[a]All items were in single-serve packaging unless noted
[b]Calories for (x2) items are reported as such in the total counts
[c]Due to availability, salad options changed for the last 10 subjects

Table S2. Full statistical results for BMI and gender as predictors of caloric and macronutrient intake during baseline, nighttime TSD, and daytime TSD. **Bold** indicates significant values.

	**Baseline** *18:00–23:59*		**Nighttime TSD** *00:00–05:59*		**Daytime TSD** *06:00–11:59*	
BMI	F	*p*	F	*p*	F	*p*
Calories	2.80	0.10	2.56	0.12	1.96	0.17
Calories From Fat	0.72	0.40	1.96	0.17	1.65	0.21
Fat	0.62	0.44	1.92	0.17	1.57	0.22
Carbohydrates	3.48	0.07	1.68	0.20	1.44	0.24
Protein	0.90	0.35	**4.63**	**0.04**	1.47	0.23
Sugar	**5.66**	**0.02**	1.20	0.28	0.27	0.61
Gender						
Calories	1.69	0.20	0.00	0.97	0.09	0.76
Calories from Fat	0.00	0.98	0.20	0.66	0.63	0.43
Fat	0.00	0.98	0.19	0.67	0.69	0.41
Carbohydrates	3.33	0.08	0.21	0.65	2.12	0.15
Protein	3.13	0.08	1.37	0.25	1.55	0.22
Sugar	**4.81**	**0.03**	0.28	0.60	3.83	0.06

Table S3. Full statistical results for the partial correlations between KSS scores and total caloric and macronutrient intake for each TSD period.

	**Baseline KSS** *18:00–23:59*		**Nighttime TSD KSS** *00:00–05:59*		**Daytime TSD KSS** *06:00–11:59*	
	r	*p*	r	*p*	r	*p*
Calories	0.06	0.72	-0.04	0.80	0.10	0.53
Calories From Fat	0.05	0.73	-0.13	0.41	-0.04	0.78
Fat	0.05	0.75	-0.13	0.40	-0.05	0.74
Carbohydrates	0.05	0.77	0.03	0.86	0.16	0.30
Protein	0.13	0.39	-0.03	0.87	0.10	0.52
Sugar	0.01	0.93	0.06	0.72	0.18	0.24

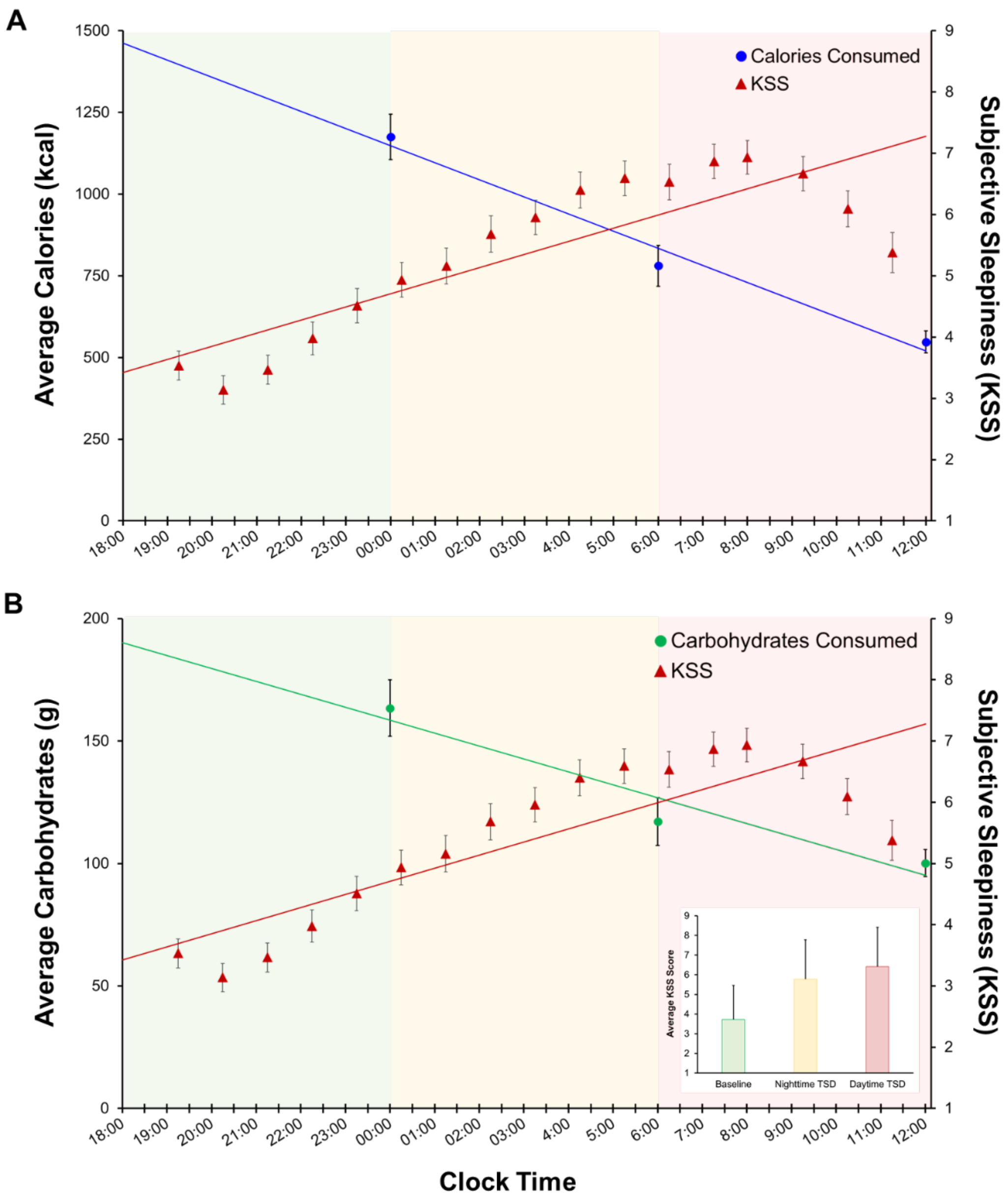

Figure S1. Regression models of mean (± SE) **(A)** total calories consumed (left axis) and subjective sleepiness ratings (right axis) and **(B)** total carbohydrates consumed (left axis) and subjective sleepiness ratings (right axis) across the sleep deprivation period. Inset: mean ± SE KSS scores for each of the three TSD periods. Shaded areas represent the three TSD periods. Green: baseline; yellow: nighttime TSD; red: daytime TSD. KSS: Karolinska Sleepiness Scale; TSD: total sleep deprivation.

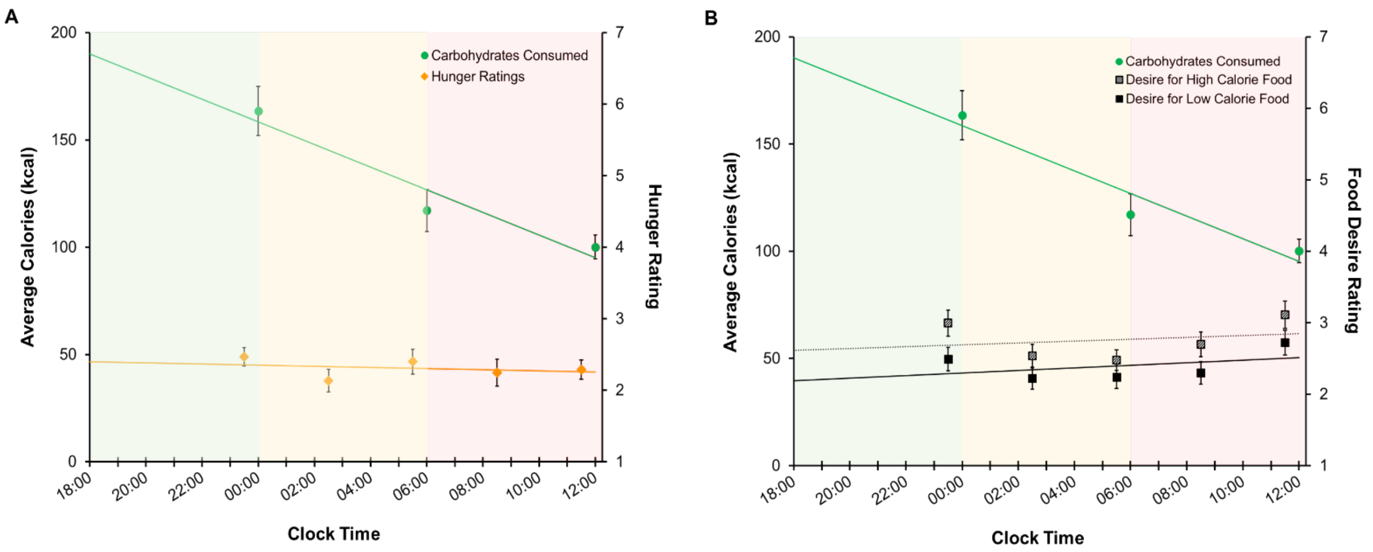

Figure S2. Regression models of mean (± SE) **(A)** carbohydrates consumed (left axis) and subjective hunger ratings (right axis) and **(B)** carbohydrates consumed (left axis) and food desire ratings (right axis) across the sleep deprivation period. Shaded areas represent the three TSD periods. Green: baseline; yellow: nighttime TSD; red: daytime TSD. TSD: total sleep deprivation.
